# Thermoresponsive Zinc TetraPhenylPorphyrin Photosensitizer/Dextran Graft Poly(N-IsoPropylAcrylAmide) Copolymer/Au Nanoparticles Hybrid Nanosystem: Potential for Photodynamic Therapy Applications

**DOI:** 10.3390/nano12152655

**Published:** 2022-08-02

**Authors:** Oleg A. Yeshchenko, Nataliya V. Kutsevol, Anastasiya V. Tomchuk, Pavlo S. Khort, Pavlo A. Virych, Vasyl A. Chumachenko, Yulia I. Kuziv, Andrey I. Marinin, Lili Cheng, Guochao Nie

**Affiliations:** 1 Physics Department, Taras Shevchenko National University of Kyiv, 60 Volodymyrska Str., 01601 Kyiv, Ukraine; nastiona30@gmail.com (A.V.T.); mirason111@gmail.com (P.S.K.); 2 Chemistry Department, Taras Shevchenko National University of Kyiv, 60 Volodymyrska Str., 01601 Kyiv, Ukraine; kutsevol@ukr.net (N.V.K.); sphaenodon@ukr.net (P.A.V.); chumachenko_va@ukr.net (V.A.C.); garaguts.yulia.fox@gmail.com (Y.I.K.); 3 Institute Charles Sadron, 23 Rue du Loess, 67200 Strasbourg, France; 4 Problem Research Laboratory, National University of Food Technology, 68 Volodymyrska Str., 01601 Kyiv, Ukraine; andrii_marynin@ukr.net; 5 Guangxi Universities Key Lab of Complex System Optimization and Big Data Processing, Yulin Normal University, Yulin 537000, China; chenglili0428@163.com

**Keywords:** thermoresponsive polymer, gold nanoparticles, photosensitizer, hybrid nanosystem, plasmon enhancement, singlet oxygen photogeneration, photodynamic therapy

## Abstract

The thermoresponsive Zinc TetraPhenylPorphyrin photosensitizer/Dextran poly (N-isopropylacrylamide) graft copolymer/Au Nanoparticles (ZnTPP/D-g-PNIPAM/AuNPs) triple hybrid nanosystem was synthesized in aqueous solution as a nanodrug for potential use in thermally driven and controlled photodynamic therapy applications. The aqueous solution of the nanosystem has demonstrated excellent stability in terms of aggregation and sedimentation several days after preparation. Optimal concentrations of the components of hybrid nanosystem providing the lowest level of aggregation and the highest plasmonic enhancement of electronic processes in the photosensitizer molecules have been determined. It has been revealed that the shrinking of D-g-PNIPAM macromolecule during a thermally induced phase transition leads to the release of both ZnTPP molecules and Au NPs from the ZnTPP/D-g-PNIPAM/AuNPs macromolecule and the strengthening of plasmonic enhancement of the electronic processes in ZnTPP molecules bound with the polymer macromolecule. The 2.7-fold enhancement of singlet oxygen photogeneration under resonant with surface plasmon resonance has been observed for ZnTPP/D-g-PNIPAM/AuNPs proving the plasmon nature of such effect. The data obtained in vitro on wild strains of *Staphylococcus aureus* have proved the high potential of such nanosystem for rapid photodynamic inactivation of microorganisms particular in wounds or ulcers on the body surface.

## 1. Introduction

Photodynamic therapy (PDT) is a promising method of treatment that can be used to solve a number of problems, from the destruction of bacteria and viruses to the treatment of cancer. This method is based on the use of three factors: light, a special light-sensitive substance—photosensitizer (PS) and oxygen from the environment to generate cytotoxic oxygen or free radicals [1,2]. Although this technique is now most widely used to treat dermatological diseases and various skin malignancies, its prospects in the destruction of various pathogens cannot be ignored, especially given the permanent emergence of new, antibiotic-resistant bacterial strains [3,4]. The main advantage of antibacterial PDT (APDT) over conventional antibiotics is that the bacteria do not show resistance to PDT even after repeated sessions of treatment.

For advances in nanobiology and nanomedicine, there is an urgent need for new hybrid functional materials based on biocompatible polymers [5,6,7,8,9,10]. A variety of biocompatible water-soluble polymers can be used to increase the bioavailability of a variety of drugs, as well as to improve drug pharmacokinetics, such as controlled delivery of drugs directly into cells and their controlled release [11]. Polymers can be used as an effective matrix for in situ synthesis of metal nanoparticles (NPs) with narrow size distribution [12,13,14,15], preventing the aggregation of NPs. An encapsulation of photosensitizers in a polymer matrix avoids aggregation of hydrophobic photosensitizer molecules, which significantly increases their photodynamic efficiency in biological media [16].

Recently, thermoresponsive polymers have attracted much attention from researchers, since such polymers are the basis for the creation of locally temperature-controlled nanoactuators [17,18,19]. In particular, poly (N-isopropylacrylamide) (PNIPAM) were used as a potential basis for the fabrication of hybrid nanosystems for applications in biology and medicine [20]. In aqueous phase, PNIPAM undergoes a phase LCST transition (lower critical solution temperature) at 32 °C from hydrophilic to hydrophobic phase, which leads to a sharp shrinking of the polymer molecule [21,22,23]. Such temperature induced shrinking can be used for the controlled release (at a given temperature) of certain molecules (e.g., drugs) initially bound to the PNIPAM macromolecule [24]. Additionally, a temperature-induced change (decrease) in the distance between molecules and plasmonic nanoparticles (NPs) leads to a change (increase) in the strength of molecule–NP coupling. This, in principle, may change the magnitude of the plasmon enhancement of electronic processes in molecules, such as absorption, emission and scattering of light, photocatalysis, generation of singlet oxygen, etc. [25,26,27,28]. The use of PNIPAM with a star-like structure allows for an increase the phase transition point by approximately 2–4 °C compared to the corresponding temperature of PNIPAM with a linear structure [26]. In addition, the Au and Ag NPs grown in situ in a branched polymer in aqueous solution have significantly higher stability than NPs grown in a linear polymer [29]. The temperature and related laser-induced phase transitions and the possibility of changing its parameters for hybrid nanosystems based on PNIPAM with a star structure (D-g-PNIPAM) and Au (Ag) NPs was shown in recent works [30,31,32,33].

Porphyrin and its derivatives are the promising photosensitizers used in particular for antibacterial and antitumor therapy [34,35]. Their advantages include high stability, efficient absorption of visible light, low dark toxicity, long life in the triplet state, highly efficient photogeneration of singlet oxygen and ease of their modification. However, there is a serious problem—the vast majority of porphyrins are hydrophobic, and therefore in living organisms they have a tendency to aggregation, which significantly reduces their photodynamic efficiency [35]. In recent years, metal porphyrins have attracted considerable attention. The PDT activity of porphyrin metal complexes depends on the type of metal due to the paramagnetic effect [36]. Zinc is added to the porphyrin ring to provide ring stability and to maintain the pronounced photodynamic efficiency of the porphyrin based PS. Such porphyrin metal complexes, e.g., zinc tetraphenylporphyrin (ZnTPP), are similar to natural porphyrin and are widely used in biology and medicine. The presence of Zn atom in the porphyrin ring has been reported to reduce mitochondrial binding and promote cell membrane binding due to complexation with phospholipid phosphate groups, which enhances the PDT efficiency [37].

Gold nanoparticles (Au NPs) are widely used in many biological applications. They are less toxic than Ag NPs [38,39]. Recently, the high antitumor efficiency has been demonstrated for some gold-based compounds [40,41]. A well-known approach to enhance the various electronic processes (light absorption, fluorescence, Raman scattering, photocatalysis, etc.) in the molecules is to place the molecules near the plasmonic metal nanoparticles or nanostructures [25,26,27,28]. In such nanoparticles, the localized surface plasmon resonance (LSPR) is excited by an external light, as a result, there is a significant increase in the electromagnetic field strength in the vicinity of plasmonic NPs, causing an increased optical response of the molecules located in the enhanced field area. Accordingly, the plasmon-enhanced light absorption by PS molecule located near the metal NP in a hybrid nanosystem would result in a more efficient photogeneration of singlet oxygen, therefore, an increase in the efficiency of PDT of the nanosystem [18,42,43,44,45]. In addition, small Au NPs have antibacterial activity against pathological bacteria due to high ability to penetrate the cell [46]. Another phenomenon with a plasmonic nature that can be used for PDT purposes is the photo-induced heating of metal NPs, plasmon heating. Due to an extremely low quantum yield of fluorescence of metal NPs, almost all of the light energy absorbed by the nanoparticle is converted to thermal energy. As a result, metal NPs act as highly efficient local nanoheaters. The effect of plasmon heating has a resonant character, i.e., it becomes strongest under the resonance of the frequencies of exciting light and LSPR in metal NP [47,48,49]. Thus, plasmon heating of metal NPs can be efficiently used for the photothermal therapy of cancer and bacterial diseases [50,51,52,53].

**Figure 1:**
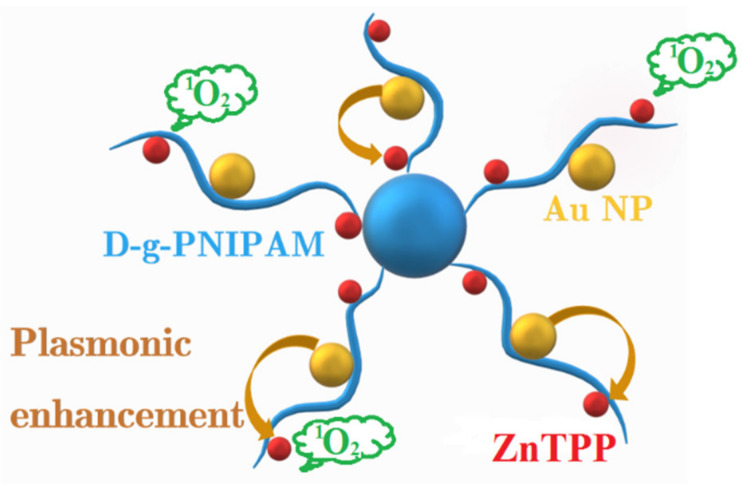
Scheme representing the structure of ZnTPP/D-g-PNIPAM/AuNPs hybrid macromolecule as well as the plasmon enhancement of singlet oxygen generation.

Here, we present the results of the chemical synthesis, the size and morphological characteristics, spectroscopic properties and APDT activity of the aqueous solution of ZnTPP/D-g-PNIPAM/AuNPs triple hybrid nanosystem. This hybrid nanosystem contains zinc tetraphenylporphyrin (ZnTPP) photosensitizer, thermoresponsive dextran poly (N-isopropylacrylamide) graft copolymer (D-g-PNIPAM) and Au NPs. Our work demonstrates that ZnTPP/D-g-PNIPAM/AuNPs nanosystem exhibits a significant plasmon enhancement of the singlet oxygen photogeneration, see scheme in Figure 1. ZnTPP/D-g-PNIPAM/AuNPs shows a high potential for thermally driven and controlled photodynamic rapid inactivation of microorganisms.

## 2. Experimental

### 2.1. Fabrication of ZnTPP/D-g-PNIPAM and ZnTPP/D-g-PNIPAM/AuNPs Hybrid Nanosystems

The procedures of synthesis and structural peculiarities of D-g-PNIPAM copolymers were reported previously. [14,30,31]. Initially, the copolymer with star-like structure containing dextran core with molecular weight *M_w_* = 70 × 10^4^ g/mol and 15 PNIPAM grafts was synthesized. The molecular parameters of D-g-PNIPAM are as follows: the molecular weight parameters *M_v_* = 1.03 × 10^6^ g/mol and *M_n_* = 0.67 × 10^6^ g/mol; *M_v_*/*M_n_* = 1.52. The reduction of Au ions took place in a mixture of aqueous solutions of the D-g-PNIPAM polymer as a matrix and HAuCl_4_ as a source of gold. The detailed description of synthesis and characterization of D-g-PNIPAM/Au NPs hybrid macromolecules in aqueous solution has been reported [30,31].

The ZnTPP powder was dissolved in ethanol at 40 °C. The obtained stock ZnTPP ethanol solution had concentration of 0.1 g/L. The aqueous stock solutions of D-g-PNIPAM (c_D-g-PNIPAM_ = 1 g/L) and D-g-PNIPAM/AuNPs (c_D-g-PNIPAM_ = 0.8696 g/L and c_Au_ = 0.8529 g/L) were diluted by water at 20 °C to obtain solutions with different concentrations. Finally, 0.03 mL of ZnTPP ethanol solution was mixed with 2.97 mL of diluted aqueous solutions of D-g-PNIPAM or D-g-PNIPAM/AuNPs. As a result, we obtained the aqueous solutions of ZnTPP/D-g-PNIPAM or ZnTPP/D-g-PNIPAM/AuNPs nanocomposite contained 0.001 g/L of ZnTPP and various concentrations of D-g-PNIPAM and/or Au.

### 2.2. Morphology and Size Characterization by TEM and DLS

The transmitted electron microscopy (TEM) measurements were performed by CM12 (FEI) microscope, and images were acquired using a Megaview SIS camera. Due to the much lower contrast of D-g-PNIPAM macromolecules compared to Au NPs, polymer macromolecules and ZnTPP molecules and aggregates are not visible on the TEM images. Therefore, the size of the studied nanosystems in solution were determined by the dynamic light scattering (DLS) method. The hydrodynamic particle size distribution (PSD) of the nanosystems in water was measured by DLS method on a Zetasizer Nano-ZS90 (Malvern Panalytical) which was equipped with a 5 mW He-Ne laser operating at 633 nm. The scattered light was detected at the angle of 173°. PSD were obtained from autocorrelation functions calculated by nonnegative truncated singular value decomposition method [54]. The DLS measurements were made in a temperature range including the LCST point. The PSD were measured at various time points (1 min—7 days) after mixing of ZnTPP ethanol solution with water, D-g-PNIPAM and D-g- PNIPAM/AuNPs aqueous solutions.

### 2.3. Absorption and Fluorescence Spectroscopy

The absorption spectra were measured with a Cary 60 UV-VIS spectrophotometer (Agilent). The fluorescence (FL) spectra were measured with a Shimadzu RF-6000 spectrofluorophotometer (Shimadzu) with an excitation wavelength at 421 nm. The solution samples were placed in 1 cm × 1 cm × 4 cm quartz cell. The spectra were measured in a temperature range including the LCST point. The absorption and FL measurements were carried out 1 day after mixing of ZnTPP ethanol solution with water, D-g-PNIPAM and D-g- PNIPAM/AuNPs aqueous solutions. The FL anisotropy were measured as $r=\frac{I_{VV}-GI_{VH}}{I_{VV}+2GI_{VH}}$, where $I_{ij}$ is the FL intensity, ij indices denote an orientation of polarizers before and after the sample respectively (V– vertical, H– horizontal), $G=I_{HV}/I_{HH}$ is the grating factor.

### 2.4. Biological Experiments

The antibacterial activity of the aqueous solutions of ZnTPP, ZnTPP/D-g-PNIPAM and ZnTPP/D-g-PNIPAM/AuNPs on wild strains of *Staphylococcus aureus* (*S. aureus*) was investigated. Bacteria were isolated on yolk-salt agar containing: meat–peptone agar—70%, sodium chloride—10%, yolk emulsion in 0.9% NaCl—20%, pH 7.3. The antibacterial activity of the nanocomposites was tested in a liquid medium of Mueller–Hinton №2 (g/L): casein hydrolysate—17.5, bull heart hydrolysate—2, water-soluble starch—1.5, pH—7.3. A suspension of *S. aureus* was prepared, then divided into 1 mL aliquots and incubated at 37 °C for 20 min, for the microorganisms to adapt to the changed conditions. The systems which were used in the investigations were also sensitive to temperature changes. Therefore, the study was performed at 28, 31, 33, 35, and 37 °C. The antibacterial activity of the nanocomposites was evaluated against control tubes that were under similar conditions. The LED-device Lika-LED (Photonics Plus) was used as a visible light source at 420 and 530 nm. The dose of irradiation was in the range of 3–18 J/mL with steps of 3 J/mL, the irradiation power was 0.1 J/s. The suspension was irradiated by light for 15 min after the addition of nanocomposites. The maximum duration of nanocomposites incubation in a suspension of *S. aureus* was not exceed 20 min.

The colony-forming units (CFU) number were counted in a Goryaev chamber. The microorganisms were stained for 1 min with acridine orange (concentration—3.5·10^−3^ M). The statistical analysis of the results was made by Shapiro–Wilk (*p* > 0.05) and Scheffe (ANOVA, *p* < 0.05) tests. The experiments were repeated three times.

## 3. Results and Discussion

### 3.1. Morphology and Size Study

**Figure 2:**
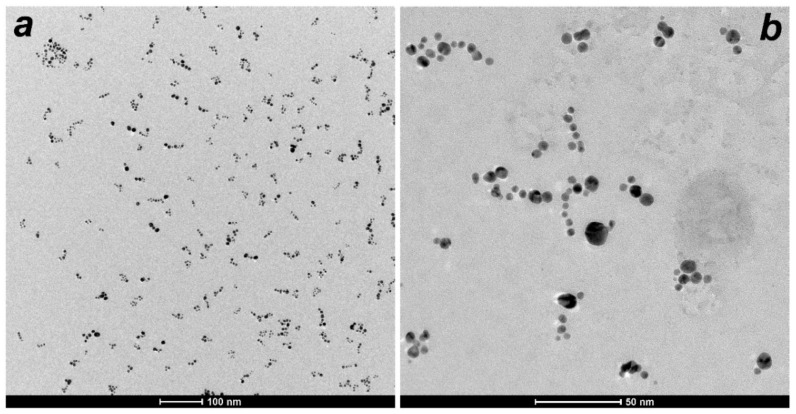
TEM images of D-g-PNIPAM/AuNPs nanohybrids at the temperature of 20 °C acquired at lower (**a**) and higher (**b**) magnification.

Typical TEM images of D-g-PNIPAM/AuNPs nanohybrids obtained at 20 °C are shown in Figure 2. Au NPs have spherical shape and a mean radius of 3 nm. Since the TEM contrast of D-g-PNIPAM polymer is significantly lower compared to Au NPs, the polymer macromolecules cannot be visualized by TEM. Meanwhile, the mean radius of the D-g-PNIPAM/AuNPs hybrid macromolecules can be estimated as the radius of Au NPs clusters on the TEM image. This estimation gives the radius of D-g-PNIPAM/AuNPs hybrid macromolecule of about 25 nm, which agrees with the DLS data (see below).

**Figure 3:**
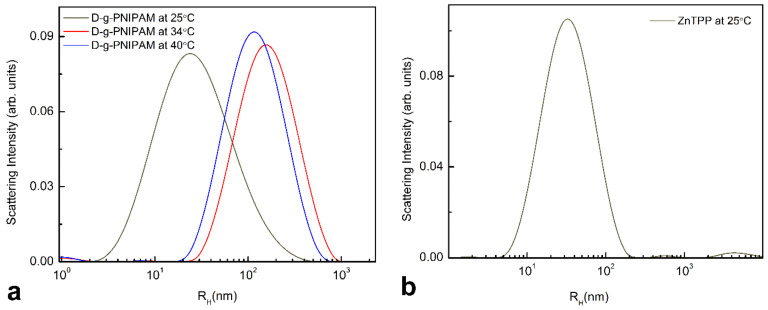
(**a**)—PSD of D-g-PNIPAM aqueous solution at temperatures lower (25 °C), equal (34 °C) and higher (40 °C) than LCST point. (**b**)—PSD of ZnTPP in ethanol mixed with water at 25 °C.

DLS measurements were performed to acquire the size of studied hybrid nanocomposites in solution. Figure 3a shows PSD change of D-g-PNIPAM during transition over LCST region at heating. The PNIPAM hydrodynamic radius (corresponding to PSD maximum) is *R_h_^max^* = 25 nm at 25 °C. At heating, D-g-PNIPAM macromolecules formed aggregates with *R_h_^max^* = 160 nm at LCST temperature of 34 °C. At subsequent heating to 40 °C higher than LCST, the D-g-PNIPAM aggregates decrease in size to 115 nm. Figure 3b demonstrates the PSD for ZnTPP in aqueous solution. In aqueous solution, the ZnTPP molecules form aggregates with a mean radius of 32 nm. It is due to hydrophobic nature of ZnTPP.

**Figure 4:**
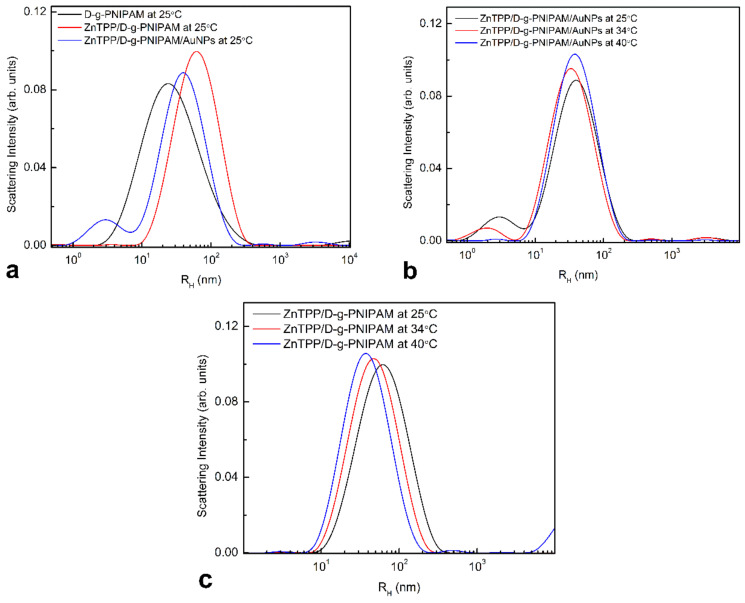
PSD of different solutions at 25 °C for comparison (**a**); triple system of ZnTPP/D-g-PNIPAM/AuNPs at 25, 34 and 40 °C (**b**); ZnTPP/D-g-PNIPAM at 25, 34 and 40 °C (**c**).

As we can see on Figure 4a, at room temperature (25 °C) the ZnTPP addition into aqueous solutions of D-g-PNIPAM and D-g-PNIPAM/AuNPs influences greatly on the average size of the respective macromolecules. Namely, for double-component ZnTPP/D-g-PNIPAM nanosystem, the average radius *R_h_^max^* shifts to 61 nm which is more than two times larger than for single-component one (D-g-PNIPAM). Size distribution of ZnTPP/D-g-PNIPAM/AuNPs has two-peaks. Those are high-intensity peak at 39 nm corresponding to ZnTPP/D-g-PNIPAM/AuNPs three-component nanosystem and low-intensity but still important peak at 3 nm corresponding to small AuNPs. Thus, hybrid macromolecules are expanded comparing to bare D-g-PNIPAM ones.

PSD of the three-component system at different temperatures (Figure 4a) shows that the main high intensity peak shifts towards smaller radius from 39 to 33 nm as the temperature rises from room 25 °C to LCST point at 34 °C. With further temperature increase, this peak shifts back to 39 nm. The observed size variation at temperature induced LCST transition is due to the shrinking of PNIPAM chains in ZnTPP/D-g-PNIPAM/AuNPs hybrid nanosystem. Such effect has been investigated for D-g-PNIPAM based multi-component hybrid nanosystems in our recent works [14,30,31,32,33]. It is important that the presence of additional components in D-g-PNIPAM based hybrid macromolecules prevents the system from aggregation at LCST transition which was observed for bare D-g-PNIPAM, see Figure 3a. This effect was explained previously [14]. Indeed, as we can see from Figure 4c, the same process takes place during heating of ZnTPP/D-g-PNIPAM system in temperature range of 25–40 °C, when the radius of ZnTPP/D-g-PNIPAM scatterers decreases gradually from 61 to 38 nm and no aggregation has been observed.

We also studied possible aging effect of the ZnTPP/D-g-PNIPAM/AuNPs nanosystem. No PSD changes were found during the period of observation up to 7 days and at the temperature variation in the temperature range of 25–40 °C, as shown in Figure S1a–c. Moreover, storage of the sample did not lead to any change of PSD at LCST transition. At 40 °C, we still can see well-defined and reproducible AuNPs peak at 2–3 nm despite low intensity of this mode, as shown in Figure S1d.

### 3.2. Absorption and Fluorescence Spectroscopy

#### 3.2.1. Spectral Manifestations of ZnTPP Binding to D-g-PNIPAM and PNIPAM/AuNPs

Light absorption spectroscopy was performed on a solution of the photosensitizer ZnTPP in ethanol, a mixture of ZnTPP ethanol solution with water, aqueous solutions of polymer D-g-PNIPAM and hybrid nanosystem D-g-PNIPAM/AuNPs, Figure S2. Hereinafter, a mixture of ZnTPP ethanol solution with water and aqueous solutions of D-g-PNIPAM and D-g-PNIPAM/AuNPs will be referred to as aqueous solutions of ZnTPP, ZnTPP/D-g-PNIPAM and ZnTPP/D-g-PNIPAM/AuNPs, respectively. The absorption spectrum of ZnTPP in ethanol (Figure S2) has a structure typical for porphyrins in organic solvents. Namely, the spectrum contains low-intensity low-energy (530–620 nm) Q bands and intense high-energy (380–440 nm) B (Soret) band [55,56,57,58]. The aqueous solution of ZnTPP/D-g-PNIPAM/AuNPs has a LSPR absorption band of AuNPs with a maximum at 520 nm, Figure S2. The respective LSPR band is clearly seen in absorption spectrum of the reference aqueous solution of D-g-PNIPAM/AuNPs (Figure S2) that proves its plasmonic nature. The D-g-PNIPAM polymer absorption and FL spectra are in UV range at wavelengths shorter than 250 nm, which is outside the spectral range relevant to this work.

The FL spectra were also measured for ZnTPP ethanol solution, mixtures of ZnTPP ethanol solution with water, and aqueous solutions of D-g-PNIPAM and D-g-PNIPAM/AuNPs, Figure S3. The FL spectrum of ZnTPP ethanol solution (Figure S3) has a structure typical for porphyrins in organic solvents. There are the high-energy (602 nm) F_00_ and low-energy (655 nm) F_01_ bands [56,57] in the FL spectrum.

Mixing ethanol solution of ZnTPP with water causes the significant changes in the shape and intensity of absorption and FL spectra, Figures S2 and S3. Such changes originate from the aggregation of hydrophobic ZnTPP molecules in the water. However, the mixing ZnTPP ethanol solution with D-g-PNIPAM and especially with D-g-PNIPAM/AuNPs aqueous solutions leads to reverse changes in absorption and FL spectra. Most probably, this is due to the binding of ZnTPP molecules to D-g-PNIPAM polymer and especially to D-g-PNIPAM/AuNPs nanohybrids. The binding increases the solubility of ZnTPP in water. It reduces the size of ZnTPP aggregates that is in full agreement with the DLS data, Figure 4a. The absorption and FL spectra changes are discussed in detail in Supplementary Materials.

Accordingly, based on the fact of essential transformations of absorption and FL spectra occurring at mixing, we conclude that ZnTPP molecules bind to D-g-PNIPAM and D-g-PNIPAM/AuNPs macromolecules, and the binding is different in the presence and absence of AuNPs. Such conclusion is also proved by the FL anisotropy measurement data, which are given and discussed below. We also emphasize that, in the aqueous media, the aggregation of ZnTPP is the weakest in the solution of ZnTPP/D-g-PNIPAM/AuNPs. Therefore, it can be expected that the biological activity of the ZnTPP photosensitizer will be higher in the ZnTPP/D-g-PNIPAM/AuNPs aqueous solution.

#### 3.2.2. Absorption and Fluorescence of ZnTPP/D-g-PNIPAM and ZnTPP/D-g-PNIPAM/AuNPs Nanosystems: Concentration Effects

The peculiarities of the interaction of ZnTPP molecules with D-g-PNIPAM and Au NPs in hybrid macromolecules have been revealed by the study of the impact of concentrations of photosensitizer, polymer and gold on the optical spectra of the nanohybrids. The relationship of the absorption and FL spectra of ZnTPP in ethanol and water with its concentration was studied in our recent works [59,60]. The dependence of ZnTPP FL intensity of ethanol solution on ZnTPP concentration was found to be non-monotonic, with a maximum FL intensity at ZnTPP concentration 0.005 g/L. Such non-monotonic dependence is caused by aggregation of ZnTPP molecules at high concentrations. Similar to ZnTPP in ethanol, the dependence of ZnTPP FL intensity in aqueous solution on ZnTPP concentration is also non-monotonic, with a maximum FL intensity at ZnTPP concentration 0.0025 g/L. The lower concentration for ZnTPP aqueous solution at the maximum FL intensity is due to the hydrophobicity of PS molecules, which promotes aggregation. Proceeding from this data, for further measurements the ZnTPP concentration of 0.001 g/L was chosen. This concentration corresponds to the middle of the growing linear region of dependence, i.e., fairly low concentrations at which aggregation is quite slight.

**Figure 5:**
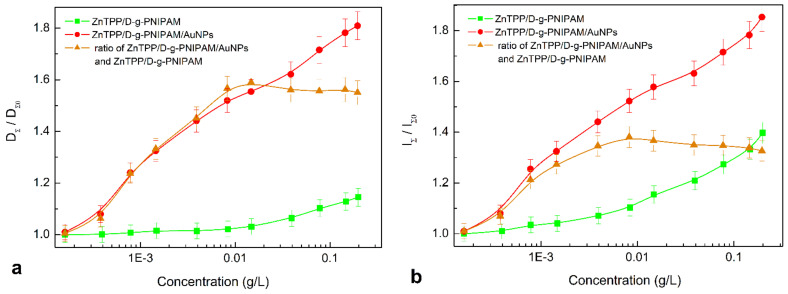
The dependence of normalized total optical density (**a**) and FL total intensity (**b**) of ZnTPP in ZnTPP/D-g-PNIPAM (squares) and ZnTPP/D-g-PNIPAM/AuNPs (circles) aqueous solutions on the concentration of polymer and gold respectively. Triangles—the dependence of ratio of optical densities and, respectively, FL intensities of ZnTPP/D-g-PNIPAM/AuNPs and ZnTPP/D-g-PNIPAM on the concentration of gold characterizing an impact of Au NPs on ZnTPP FL in the hybrid nanosystem. The optical density and FL intensity are normalized to the respective values for ZnTPP in aqueous solution. ZnTPP concentration is 0.001 g/L.

Next, the effect of polymer and Au concentration on magnitude of absorption (total optical density—integrated over the entire absorption spectrum) and FL (FL total intensity—integrated over the entire FL spectrum) of ZnTPP in aqueous solutions of ZnTPP/D-g-PNIPAM and ZnTPP/D-g-PNIPAM/AuNPs nanohybrids was investigated, Figure 5a,b respectively. The optical density and FL intensity at different concentrations were normalized against ZnTPP in aqueous solution. As shown in Figure 5, the concentration dependences of the optical density and FL intensity are the similar. Therefore, further we analyze only the concentration dependence of FL intensity. The dependences of ZnTPP FL intensity on D-g-PNIPAM concentration for ZnTPP/D-g-PNIPAM, and on Au concentration for ZnTPP/D-g-PNIPAM/AuNPs nanosystems are monotonically increasing in the whole studied range of concentrations, Figure 5. Proceeding from mentioned above, such dependences can be rationalized as a consequence of the fact that both the D-g-PNIPAM and D-g-PNIPAM/AuNPs inhibit the aggregation of ZnTPP molecules in aqueous medium. Herewith, this effect is significantly stronger for nanosystems containing Au NPs. In order to highlight the impact of Au NPs concentration on the ZnTPP FL intensity, the ratio of concentration dependences for ZnTPP/D-g-PNIPAM/AuNPs and ZnTPP/D-g-PNIPAM was calculated and plotted against ZnTPP concentration (Figure 5, triangles). It gives the dependence of the FL plasmon-enhancement factor on the gold concentration. It shows that the intensity of FL increases in 1.4 times in the concentration range of 0–0.008 g/L, reaches a maximum, and then decreases to 1.33 with a further increase of concentration to 0.2 g/L. The observed plasmonic enhancement of FL of photosensitizer molecules in the ZnTPP/D-g-PNIPAM/AuNPs nanosystem indicates that ZnTPP molecules and Au NPs are quite closely located inside the polymer macromolecule, which is due to binding of ZnTPP molecules to D-g-PNIPAM/AuNPs hybrid macromolecules.

We analyze the physical mechanisms of the influence of Au NPs on FL of ZnTPP in hybrid macromolecules. By changing the Au NPs concentration, we thereby change the mean ZnTPP–AuNP distance inside the D-g-PNIPAM macromolecule. The distance change affects the strength of coupling of Au NPs and ZnTPP molecules [61,62,63,64,65,66]. The strength of coupling depends strongly on the overlap of LSPR in metal NP and the electronic energy spectrum of the fluorophore molecule. The coupling is stronger at shorter distances and higher spectral overlap. It is mentioned above that ZnTPP molecules and Au NPs are closely located inside the hybrid macromolecule network. In addition, there is a significant overlap of the LSPR absorption band of the Au NPs with the absorption and FL spectra of ZnTPP molecules, Figures S2 and S3. Thus, a strong coupling of Au NPs with ZnTPP molecules in ZnTPP/D-g-PNIPAM/AuNPs nanosystem is highly expected.

It is well known that there are two competing physical mechanisms for the donor (dye molecule-fluorophore)—acceptor (metal NP) pair that affect the FL intensity of the dye. The first mechanism is the plasmon enhancement. It strengthens with decreasing distance between the molecule and the metal NP [61,62,63,64,65,66,67]. The amplitude of plasmon field depends on the distance from the metal NP as $E_{sp}\propto R^{-3}$ [66,67]. The second mechanism is the non-radiative Förster resonant energy transfer (FRET) from the excited donor (fluorophore molecule) to the acceptor (metal NP) due to dipole-dipole interaction [61,62,63,67,68]. FRET leads to quenching of FL. The rate of FL quenching depends on the distance between the donor and acceptor as $\gamma_{FRET}\propto R^{-6}$ [68]. Such dependence limits the FRET to distances below 10 nm. The competition between plasmon enhancement and FRET quenching leads to an existence of the optimal NP-molecule distance (about 10 nm) providing the highest FL intensity [63]. At distances less than 10 nm, a small decrease in distance causes the strong FL quenching. Meanwhile, at distances larger than 10 nm, a decrease in distance causes the FL enhancement.

Thus, at lower concentrations of Au NPs in the range of 0–0.008 g/L, the ZnTPP–AuNP distance is too large for FRET. An increase in the concentration of Au NPs causes the shortening of ZnTPP–AuNP distance, which leads to the stronger plasmon enhancement of FL. At concentrations of Au NPs higher than 0.008 g/L, the ZnTPP–AuNP distance becomes short enough for FRET, which leads to FL quenching at the increase of Au NPs concentration. Therefore, the conclusion can be made that there is a certain optimal Au NPs concentration, which provides the highest plasmon enhancement of optical processes involving the ZnTPP photosensitizer molecules, in particular light absorption, fluorescence and singlet oxygen generation.

**Figure 6:**
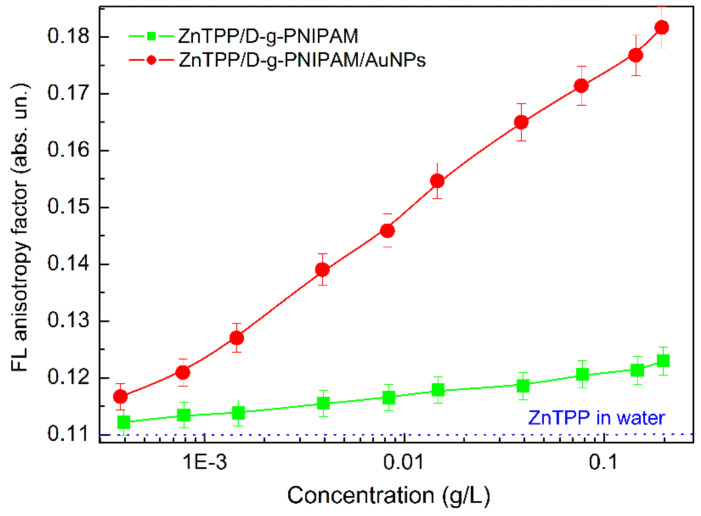
Concentration dependence of FL anisotropy for ZnTPP in aqueous solutions of ZnTPP/D-g-PNIPAM (squares) and ZnTPP/D-g-PNIPAM/AuNPs (circles). Squares and circles show the dependence on D-g-PNIPAM and AuNPs concentration respectively. Concentration of ZnTPP is 0.001 g/L. The blue dotted line corresponds to ZnTPP in water.

Assumptions of the binding of ZnTPP molecules to the D-g-PNIPAM and D-g-PNIPAM/AuNPs can be checked directly by FL anisotropy *r* measurement. The FL anisotropy indicates how constrained the molecules is in its motion. For ZnTPP molecules in ethanol solution, *r* is 0.007 that indicates the almost free motion of ZnTPP molecules in ethanol. However, for ZnTPP molecules in water, *r* is 0.110 that indicates a higher constraint of the motion of ZnTPP molecules in water. Most probably, that is due to the formation of ZnTPP aggregates. Mixing ZnTPP with an aqueous solution of D-g-PNIPAM leads to a further increase of the FL anisotropy, which increases from 0.112 to 0.123 with increasing polymer concentration in the range of 0.00039–0.198 g/L, Figure 6. Meanwhile, in ZnTPP/D-g-PNIPAM/AuNPs aqueous solution, the *r* factor increases from 0.117 to 0.182 with increasing gold concentration in the range of 0.00039–0.195 g/L, Figure 6. Thus, the obtained results prove the fact of binding of ZnTPP molecules both with D-g-PNIPAM macromolecules and D-g-PNIPAM/AuNPs hybrid macromolecules. Thus, the FL anisotropy measurement data indicate that ZnTPP molecules bind better to hybrid macromolecules containing Au NPs that is in full agreement with data obtained from absorption and FL spectra of ZnTPP/D-g-PNIPAM and ZnTPP/D-g-PNIPAM/AuNPs hybrids, Figures S2 and S3 and above relevant discussions.

#### 3.2.3. Thermally Induced Processes in ZnTPP/D-g-PNIPAM and ZnTPP/D-g-PNIPAM/AuNPs Nanohybrids

**Figure 7:**
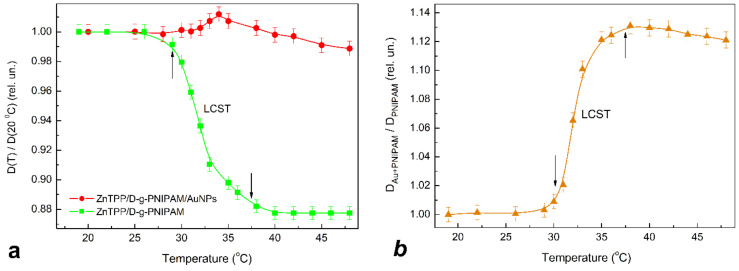
(**a**)—Dependence of total (B and Q bands) optical density on temperature for ZnTPP in aqueous solutions of ZnTPP/D-g-PNIPAM (squares) and ZnTPP/D-g-PNIPAM/AuNPs (circles) during heating. (**b**)—Dependence of the ratio of total optical densities for ZnTPP in ZnTPP/D-g-PNIPAM/AuNPs and ZnTPP/D-g-PNIPAM on temperature characterizing the impact of Au NPs on ZnTPP light absorption in ZnTPP/D-g-PNIPAM/AuNPs nanosystem. The optical density is normalized by value at 20 °C. Arrows show the LCST transition range. Concentrations: ZnTPP—0.001 g/L, D-g-PNIPAM—0.078 g/L, Au—0.077 g/L.

**Figure 8:**
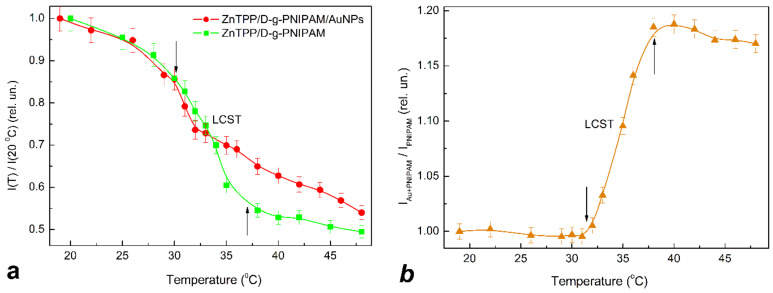
(**a**)—Temperature dependence of total FL intensity for aqueous solutions of ZnTPP/D-g-PNIPAM (squares) and ZnTPP/D-g-PNIPAM/AuNPs (circles) during heating. (**b**)—Temperature dependence of the ratio of total FL intensities for ZnTPP/D-g-PNIPAM/AuNPs and ZnTPP/D-g-PNIPAM characterizing the impact of Au NPs on FL of ZnTPP in ZnTPP/D-g-PNIPAM/AuNPs nanosystem. The FL intensity is normalized by value at 20 °C. Arrows show the LCST transition range. Concentrations: ZnTPP—0.001 g/L, D-g-PNIPAM—0.078 g/L, Au—0.077 g/L.

Since PNIPAM is a thermoresponsive polymer, it is reasonable to expect that the LCST phase transition should cause the thermally induced processes in ZnTPP/D-g-PNIPAM and ZnTPP/D-g-PNIPAM/AuNPs systems. The respective transformations were probed by light absorption (Figure 7) and FL (Figure 8) spectroscopy of ZnTPP molecules in aqueous solutions of ZnTPP/D-g-PNIPAM and ZnTPP/D-g-PNIPAM/AuNPs. It was revealed that, during heating, passing through the LCST point leads to 1.14 times decrease of light absorption by ZnTPP in ZnTPP/D-g-PNIPAM, while the temperature dependence of light absorption by ZnTPP in ZnTPP/D-g-PNIPAM/AuNPs is non-monotonic with slightly expressed maximum at LCST point, Figure 7a. Meanwhile, passing through the LCST point during heating leads to FL quenching both for ZnTPP/D-g-PNIPAM and ZnTPP/D-g-PNIPAM/AuNPs, Figure 8a. Note that quenching is stronger for ZnTPP in ZnTPP/D-g-PNIPAM than in ZnTPP/D-g-PNIPAM/AuNPs. Indeed, the FL intensity decreased 2.02 and 1.85 times when the sample was heated from 20 to 48 °C for ZnTPP/D-g-PNIPAM and ZnTPP/D-g-PNIPAM/AuNPs respectively. The impact of temperature on the light absorption and FL is especially strong in the region of LCST point, i.e., at a temperature of about 35 °C.

The observed behavior of the temperature dependences of absorption and FL in the temperature region of LCST transition can be caused by three physical mechanisms. First one is the release of the photosensitizer molecules out from the polymer macromolecule due to its shrinking at the LCST transition. Indeed, the released ZnTPP molecules are located far from the Au NPs in the spatial areas, where the plasmonic field is slight enough to enhance the FL. Thus, the release of ZnTPP should lead to a sharp decrease of plasmonic enhancement and, correspondingly, to a decrease of light absorption and FL quenching. Second one is the occurrence of FRET occurring due to the sharp decrease of the distance between ZnTPP molecules and Au NPs remaining bound to polymer macromolecule while it is shrinking. It leads to FL quenching at LCST transition. Meanwhile, the shortening of the distance between the ZnTPP molecules and Au NPs bound to D-g-PNIPAM can also lead to the opposite process (third mechanism), namely the intensification of the plasmonic enhancement of light absorption and FL of ZnTPP molecules bound to the polymer.

**Figure 9:**
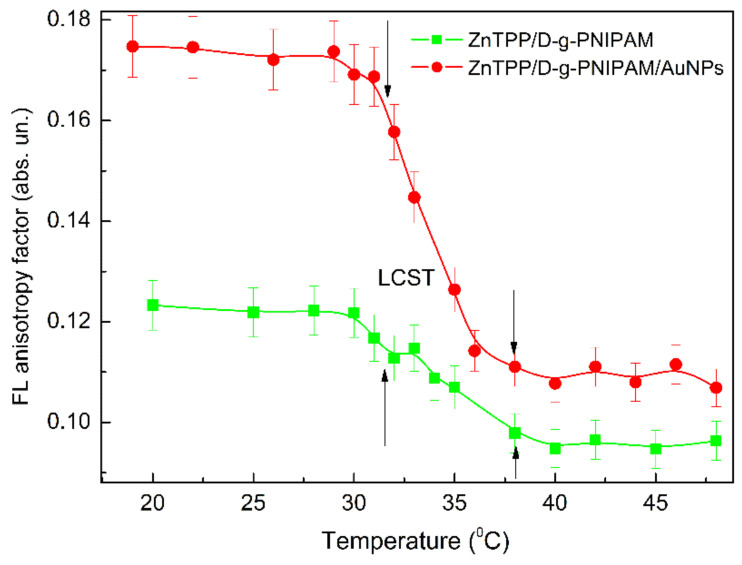
Temperature dependence of FL anisotropy factor for ZnTPP in aqueous solutions of ZnTPP/D-g-PNIPAM (squares) and ZnTPP/D-g-PNIPAM/AuNPs (circles) during heating. Arrows show the LCST transition range. Concentrations: ZnTPP—0.001 g/L, D-g-PNIPAM—0.078 g/L, Au—0.077 g/L.

In order to find out which physical mechanisms are dominant, the temperature dependence of the FL anisotropy was measured, Figure 9. It clearly shows that LCST transition leads to sharp decrease of the FL anisotropy from 0.12 to 0.09 for ZnTPP/D-g-PNIPAM and from 0.17 to 0.11 for ZnTPP/D-g-PNIPAM/AuNPs. The observed sharp decrease of the FL anisotropy proves the fact of the release of the ZnTPP molecules when the polymer macromolecule is shrinking. It is reasonable to expect that the polymer shrinking also leads to the release of the Au NPs. Thus, the conclusion can be made that both ZnTPP/D-g-PNIPAM and ZnTPP/D-g-PNIPAM/AuNPs nanosystems are promising for thermally induced and controlled drug release.

In order to compare the impact of FRET and plasmonic enhancement on the light absorption, the temperature dependence of the ratio of total (integrated spectrally over B and Q bands) optical density for ZnTPP in ZnTPP/D-g-PNIPAM/AuNPs and one in ZnTPP/D-g-PNIPAM is calculated and presented in Figure 7b. Respectively, in order to compare the impact of the FRET and plasmonic enhancement on the FL intensity, the temperature dependence of the ratio of total FL intensities for ZnTPP in ZnTPP/D-g-PNIPAM/AuNPs and ZnTPP/D-g-PNIPAM is calculated and presented in Figure 8b. These ratio dependences characterize the influence of processes involved the Au NPs on light absorption and FL of the ZnTPP molecules which are bound to the polymer. The free ejected ZnTPP molecules do not contribute to this dependence. Figure 7b and Figure 8b show that the polymer macromolecule shrinking at phase transition causes the sharp increase in light absorption and FL intensity for ZnTPP molecules bound with D-g-PNIPAM/AuNPs hybrid macromolecule, comparing to ZnTPP molecules bound with bare D-g-PNIPAM macromolecule. The observed increase in relative optical density and FL intensity is due to the strengthening of plasmonic enhancement, which was caused by the shortening of the mean distance between Au NPs and ZnTPP molecules bound to polymer macromolecule. Thus, one can conclude that the plasmonic enhancement prevails the FRET at the temperature induced phase transition at the determined optimal concentrations of ZnTPP and Au NPs. Most probably, the revealed temperature induced strengthening of plasmonic enhancement should also lead to the increase in efficiency of singlet oxygen generation in ZnTPP/D-g-PNIPAM/AuNPs at phase transition. Thus, summarizing the obtained data, we conclude that the temperature induced LCST transition in ZnTPP/D-g-PNIPAM/AuNPs nanosystem leads to the release of ZnTPP molecules and Au NPs from the macromolecule, as well as to the strengthening of plasmonic enhancement of the optical processes in ZnTPP molecules bound with the polymer macromolecules. Both these processes make the ZnTPP/D-g-PNIPAM/AuNPs nanosystem quite efficient for thermally driven and controlled PDT applications that is discussed in next Section 3.4.

### 3.3. Singlet Oxygen Photogeneration Enhancement in ZnTPP/D-g-PNIPAM/AuNPs System

**Figure 10:**
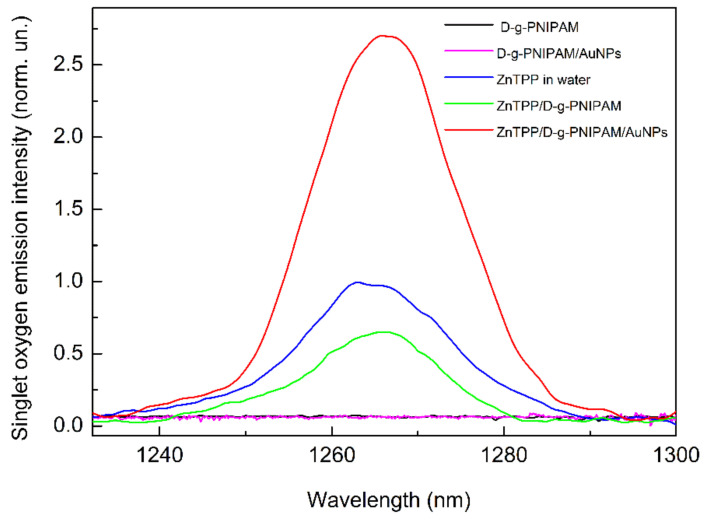
Emission spectra of singlet oxygen for ZnTPP, ZnTPP/D-g-PNIPAM and ZnTPP/D-g- PNIPAM/AuNPs aqueous solutions. The D-g-PNIPAM and D-g-PNIPAM/AuNPs aqueous solutions without ZnTPP demonstrate no emission from singlet oxygen. Excitation: 553 nm; concentrations: ZnTPP—0.001 g/L, D-g-PNIPAM—0.078 g/L, Au—0.077 g/L.

An important characteristic of the efficiency of some molecular systems for use in PDT is the efficiency of photogeneration of singlet oxygen. Figure 10 shows the measured spectra of singlet oxygen emission from aqueous solutions of ZnTPP/D-g-PNIPAM and ZnTPP/D-g-PNIPAM/AuNPs systems, as well as the reference spectra of ZnTPP, D-g-PNIPAM and D-g-PNIPAM/Au NPs aqueous solutions. One can see that the aqueous solutions of D-g-PNIPAM and D-g-PNIPAM/AuNPs without ZnTPP demonstrate no singlet oxygen emission. Meanwhile, the spectra of ZnTPP containing systems show the emission peak at 1270 nm, a characteristic for singlet oxygen. Singlet oxygen emission spectra were detected for ZnTPP, ZnTPP/D-g-PNIPAM and ZnTPP/D-g-PNIPAM/AuNPs samples under excitation at 421 nm (Sore band), 553 nm and 595 nm (range of Q bands). Under excitations at 421 nm and 595 nm, the peak intensities are approximately the same for all ZnTPP containing systems. Additionally, under 553 nm excitation of aqueous solutions of ZnTPP and ZnTPP/D-g-PNIPAM, the peak intensities are close to the corresponding values obtained under 421 nm and 595 nm excitation. However, under 553 nm excitation of the ZnTPP/D-g-PNIPAM/AuNPs solution, a considerable 2.7-fold rise in intensity of the singlet oxygen peak is observed. Considering that the wavelength of 553 nm is resonant with LSPR in Au NPs (520 nm), it is reasonable to conclude that such enhancement of photogeneration of singlet oxygen has a plasmonic nature. Thus, the observed plasmonic enhancement of singlet oxygen photogeneration by ZnTPP/D-g-PNIPAM/AuNPs nanohybrid indicates its potential for PDT purposes that has proved by our biological studies discussed in Section 3.4.

### 3.4. Photodynamic Antibacterial Activity In Vitro

**Figure 11:**
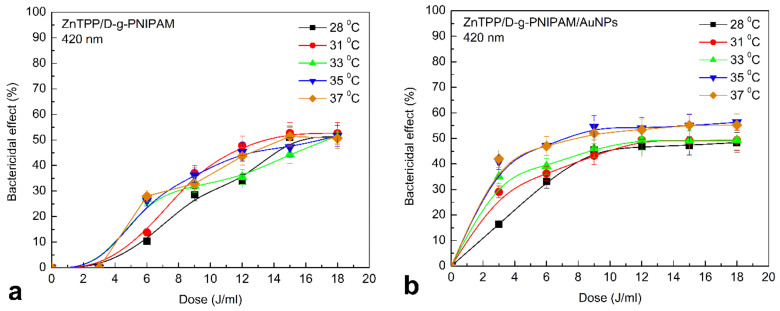
*S. aureus* inactivation at 28, 31, 33, 35, 37 °C after adding of ZnTPP/D-g-PNIPAM (**a**), ZnTPP/D-g-PNIPAM/AuNPs (**b**), and irradiation by light at 420 nm depending on the irradiation dose. Concentrations: AuNPs and D-g-PNIPAM—0.08 g/L, 0.008 g/L, 0.0008 g/L, ZnTPP—0.001 g/L. Power of light—0.1 J/s, dose of irradiation was in the range of 3–18 J/mL with 3 J/mL increment.

**Figure 12:**
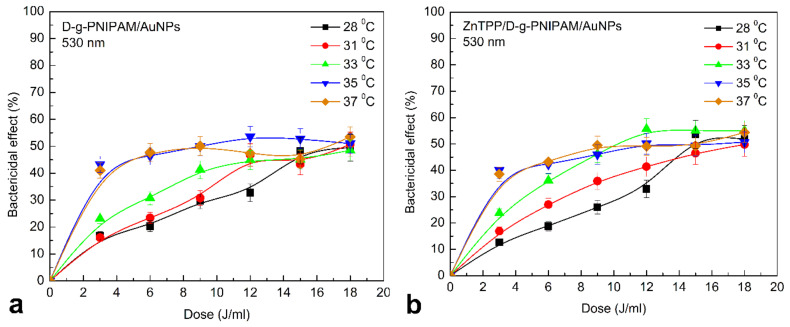
*S. aureus* inactivation at 28, 31, 33, 35, 37 °C after adding of D-g-PNIPAM/AuNPs (**a**), ZnTPP/D-g-PNIPAM/AuNPs (**b**), and irradiation by light at 530 nm depending on the irradiation dose. Concentrations: AuNPs and D-g-PNIPAM—0.08 g/L, 0.008 g/L, 0.0008 g/L, ZnTPP—0.001 g/L. Power of light—0.1 J/s, dose of irradiation was in the range of 3–18 J/mL with 3 J/mL increment.

Thermoresponsive hydrogel systems are used as injectable gel-forming matrices. In the sol phase, therapeutic agents can be included in the system, and after injection to the target tissues, the solution is transformed into a gel and serves as a source of a drug. PNIPAM is one of the most common polymers of this type. Weak cytotoxicity of D-g-PNIPAM carrier against *S. aureus* at 37 °C was detected, Figure S4. The results are consistent with the literature data [69,70]. In Figures S4–S8, Figure 11 and Figure 12, we chose the same scale on the *Y* axis for the convenience of quantitative comparison of the bactericidal effect under different conditions of biological experiments.

At 0.008 g/L concentration of D-g-PNIPAM polymer, there was no toxicity against *S. aureus*. Therefore, for further studies of the antibacterial activity of hybrid nanosystems, we chose a concentration of D-g-PNIPAM at 0.008 g/L to offset the toxic effects of the polymer and assess its response to temperature changes. LCST phase transition in D-g-PNIPAM occurs at 33–34 °C. Therefore, the copolymer is in the hydrophilic sol phase at 28 °C, and at human body temperature—in the hydrophobic gel phase. Comparison of antibacterial activity under such conditions indicates the retention of Au NPs in D-g-PNIPAM at temperatures below the LCST point, Figure S5. There was a 45–50% decrease of CFU amount at 37 °C for 0.08 and 0.008 g/L Au NPs concentrations after 25 min. Thus, one can assume that the polymer macromolecule shrinking at phase transition causes the release of Au NPs out to the solution.

Incubation of *S. aureus* suspension with 0.08 g/L D-g-PNIPAM/AuNPs and irradiation by 530 nm had a little effect on the antibacterial activity of nanocomposite at 37 °C, Figure S6. However, after reducing the concentration of D-g-PNIPAM/AuNPs to 0.008 g/L under similar conditions, the initial antibacterial activity of the nanocomposite increased by 40%.

The identified effects can be related to several factors. Probably, during shrinking of the Au NPs carrier, i.e., D-g-PNIPAM macromolecule, Au NPs are rapidly released into solution. The copolymer stabilizes AuNPs and prevents their aggregation. The high concentration of Au NPs increases the probability of their encounter and the formation of biologically inactive aggregates. Irradiation by light at 530 nm can accelerate the aggregation in the absence of a stabilizer. Some studies confirm the possibility of the aggregation under the light irradiation [31,33,71,72,73]. Reducing the D-g-PNIPAM/AuNPs concentration retains the necessary antibacterial properties when irradiated by light at 530 nm, Figure S6.

Low sensitivity of the triple ZnTPP/D-g-PNIPAM/AuNPs system to the action of 420 nm light at 37 °C was revealed, Figure S7. Only irradiation of a bacterial suspension with 0.08 g/L nanocomposite concentration contributes to a linear increase in bactericidal activity from 33% to 75% depending on dose used. Additionally, for a 0.008 g/L Au NPs concentration and 3 J/mL irradiation dose, CFU amount further decrease by 25–30%. We assume that this is due to the features of the carrier. After the phase transition, it does not facilitate the delivery of active components to bacterial cells. In addition, to inactivate the bacterial cell, it is necessary to disrupt vital structures such as DNA, plasma membrane, enzymes etc. If the nanosystems do not penetrate the cells but are adsorbed on the cell wall or capsule instead, the damage of the cells is not critical. If such interactions are indeed present, nanocomposites can be used as the sensors for micro-organisms detection [74]. Irradiation by light at 530 nm of nanocomposite ZnTPP/D-g-PNIPAM/AuNPs contributes to the change of the number of CFU similar to the results for the system D-g-PNIPAM/AuNPs, Figure S8.

The results indicate that under such conditions, the Au NPs play a decisive role in the antibacterial activity, and a photosensitizer plays the secondary role at least at used concentrations. When irradiated by light, the antibacterial activity of nanocomposites depends on temperature. The change is clear at low dose (3–6 J/mL) of light irradiation (Figure 11). There are no detectable changes in the antibacterial properties under blue light irradiation at doses higher than 6 J/mL. The presence of Au NPs helps to increase the sensitivity of the nanosystems to 420 nm light (Figure 11b). The more pronounced temperature dependence was also found for ZnTPP/D-g-PNIPAM/AuNPs. This once again proves the decisive role of Au NPs in antibacterial activity. The results obtained at the irradiation of D-g-PNIPAM/AuNPs and ZnTPP/D-g-PNIPAM/AuNPs nanosystems by light at 530 nm resonant with LSPR in Au NPs prove this assumption (Figure 12). The rise of temperature closer to the point of LCST phase transition leads to a gradual increase in antibacterial activity. The antibacterial properties were the same at 35 and 37 °C.

The obtained temperature dependencies for irradiation of nanosystems at 420 and 530 nm indicate the determining role of Au NPs in the inactivation of microorganisms. The photosensitizer plays a secondary role in this process. However, it is noticeable that under the irradiation by 420 nm light resonant with ZnTPP Soret absorption band and irradiation doses lower than 10 J/mL, the antibacterial activity of ZnTPP/D-g-PNIPAM/AuNPs is essentially higher than one of D-g-PNIPAM/AuNPs, Figure 11. Most probably, this effect is caused by the plasmonic enhancement of the singlet oxygen generation by the ZnTPP molecules located in plasmonic field of Au NPs. As we discussed above, the local plasmon field enhances the optical processes in the photosensitizer molecules located in this field. Indeed, we revealed an enhancement of ZnTPP fluorescence in ZnTPP/D-g-PNIPAM/AuNPs nanosystem with the maximal FL enhancement at Au NPs concentration of 0.008 g/L (Figure 5). Another electronic process in ZnTPP molecules, that is probably plasmon enhanced, is the singlet oxygen generation. Such suggestion is proved by following observations. Figure 11 shows that at temperatures higher than LCST point, the antibacterial activity of ZnTPP/D-g-PNIPAM/AuNPs is elevated compared to ZnTPP/D-g-PNIPAM. Indeed, the macromolecule shrinking at the LCST transition leads to a decrease in ZnTPP–AuNP distance. As a result, the ZnTPP molecules are located in stronger plasmon field that should cause the enhancement of both singlet oxygen generation and FL by ZnTPP molecules. The singlet oxygen photogeneration enhancement causes an increase of antibacterial activity. In turn, as we discussed above, the LCST transition leads to enhancement of ZnTPP FL in ZnTPP/D-g-PNIPAM/AuNPs system comparing to ZnTPP/D-g-PNIPAM, Figure 11b.

Double and triple thermoresponsive nanosystems based on D-g-PNIPAM copolymer with Au NPs and ZnTPP photosensitizer, were irradiated by small doses of light of 420 and 530 nm. As the result, the number of CFU was decreased by 50% within 20 min. This bactericidal effect is not so strong when compared with antibiotics or other antibacterial chemicals [75,76]. Despite this, the investigated nanosystems have the significant potential to be used as broad-spectrum bactericidal agents with a reduced risk of developing resistance in bacteria. The LCST phase transition in D-g-PNIPAM provides a rapid increase in biological activity of D-g-PNIPAM based nanohybrids at temperatures lower than physiological. Given the results obtained on the properties of ZnTPP/D-g-PNIPAM/AuNPs nanohybrid, it can be applied on the body surface, wounds, or ulcers for rapid inactivation of microorganisms.

## 4. Conclusions

In conclusion, we have presented the results of the synthesis, the size and morphology characteristics, spectral properties and APDT activity of the aqueous solution of ZnTPP/D-g-PNIPAM/AuNPs triple hybrid nanosystem containing zinc tetraphenylporphyrin (ZnTPP) photosensitizer, thermoresponsive dextran poly (N-isopropylacrylamide) polymer (D-g-PNIPAM) and Au nanoparticles. Spectroscopic manifestations of binding of ZnTPP molecules to D-g-PNIPAM/AuNPs macromolecules have been obtained. The ZnTPP/D-g-PNIPAM/AuNPs nanohybrid has demonstrated high morphological stability (absence of the aggregation) up to 7 days after preparation. Optimal concentrations of the components of hybrid nanosystem providing the weak aggregation and the high plasmonic enhancement of electronic processes in photosensitizer molecules have been determined as following: ZnTPP—0.001 g/L, D-g-PNIPAM—0.078 g/L, Au—0.077 g/L. The shrinking of D-g-PNIPAM macromolecule at a thermally induced LCST phase transition has been revealed to lead to Au NPs and ZnTPP molecules release from the ZnTPP/D-g-PNIPAM/AuNPs macromolecule, as well as to strengthening of plasmon enhancement of the optical processes in ZnTPP molecules bound with the polymer macromolecule. The 2.7-fold plasmon enhancement of the photogeneration of singlet oxygen under excitation resonant with LSPR in Au NPs has been demonstrated for ZnTPP/D-g-PNIPAM/AuNPs system, which indicates its potential for PDT applications. The data obtained in vitro on *Staphylococcus aureus* wild strains have proved the potential of such nanosystem for rapid inactivation of microorganisms. In particular, the significant increase of PDT based bactericidal efficiency of ZnTPP/D-g-PNIPAM/AuNPs nanohybrid at the temperatures higher than LCST transition point has been observed at irradiation doses lower than 8 J/mL. This indicates that aqueous solution of ZnTPP/D-g-PNIPAM/AuNPs nanohybrid has a potential for thermally driven and controlled PDT applications at low light irradiation doses, in particular for rapid antibacterial PDT.

## Data Availability

Data are within the article and Supplementary Materials.

## Supplementary Materials

The following supporting information can be downloaded at: https://www.mdpi.com/article/10.3390/nano12152655/s1, Aging effect on ZnTPP/D-g-PNIPAM/AuNPs nanosystem in aqueous solution from DLS measurements (Figure S1), Transformation of absorption spectrum of ZnTPP in water and aqueous solutions of hybrid nanosystems (Figure S2 and analysis), Transformation of FL spectrum of ZnTPP in water and aqueous solutions of hybrid nanosystems (Figure S3 and analysis), Remarks on the reabsorption effect on concentration dependence of FL, Additional information on photodynamic antibacterial activity of nanosystems in vitro (Figures S4–S8).
