# Supplementary material for: Thermoresponsive Zinc TetraPhenylPorphyrin Photosensitizer/Dextran Graft Poly(N-IsoPropylAcrylAmide) Copolymer/Au Nanoparticles Hybrid Nanosystem: Potential for Photodynamic Therapy Applications
Source: Nanomaterials (Basel). 2022 Aug 2;12(15):2655. doi: 10.3390/nano12152655 (PMC9370275; doi:10.3390/nano12152655)
Supplement: Supplementary file 1 [file nanomaterials-12-02655-s001.zip › nanomaterials-1804763-supplementary.pdf]

nanomaterials

Supplementary Materials

Thermoresponsive Zinc TetraPhenylPorphyrin Photosensitizer/Dextran Graft Poly(N-IsoPropylAcrylAmide) Copolymer/Au Nanoparticles Hybrid Nanosystem: Potential for Photodynamic Therapy Applications

Oleg A. Yeshchenko [1,*], Nataliya V. Kutsevol [2,3], Anastasiya V. Tomchuk [1], Pavlo S. Khort [1], Pavlo A. Virych [2], Vasyl A. Chumachenko [2], Yulia I. Kuziv [2,3], Andrey I. Marinin [4], Lili Cheng [5] and Guochao Nie [5,*]

[1] Physics Department, Taras Shevchenko National University of Kyiv, 60 Volodymyrska Str., 01601 Kyiv, Ukraine; nastiona30@gmail.com (A.V.T.); mirason111@gmail.com (P.S.K.)
[2] Chemistry Department, Taras Shevchenko National University of Kyiv, 60 Volodymyrska Str., 01601 Kyiv, Ukraine; kutsevol@ukr.net (N.V.K.); sphaenodon@ukr.net (P.A.V.); chumachenko_va@ukr.net (V.A.C.); garaguts.yulia.fox@gmail.com (Y.I.K.)
[3] Institute Charles Sadron, 23 Rue du Loess, 67200 Strasbourg, France; kutsevol@ukr.net (N.V.K.); garaguts.yulia.fox@gmail.com (Y.I.K.)
[4] Problem Research Laboratory, National University of Food Technology, 68Volodymyrska Str., 01601 Kyiv, Ukraine; andrii_marynin@ukr.net
[5] Guangxi Universities Key Lab of Complex System Optimization and Big Data Processing, Yulin Normal University, Yulin 537000, China; chenglili0428@163.com
* Correspondence: oleg.yeshchenko@knu.ua (O.A.Y.); bccu518@163.com (G.N.)

1. Aging Effect on ZnTPP/D-g-PNIPAM/AuNPs Nanosystem in Aqueous Solution from DLS Measurements

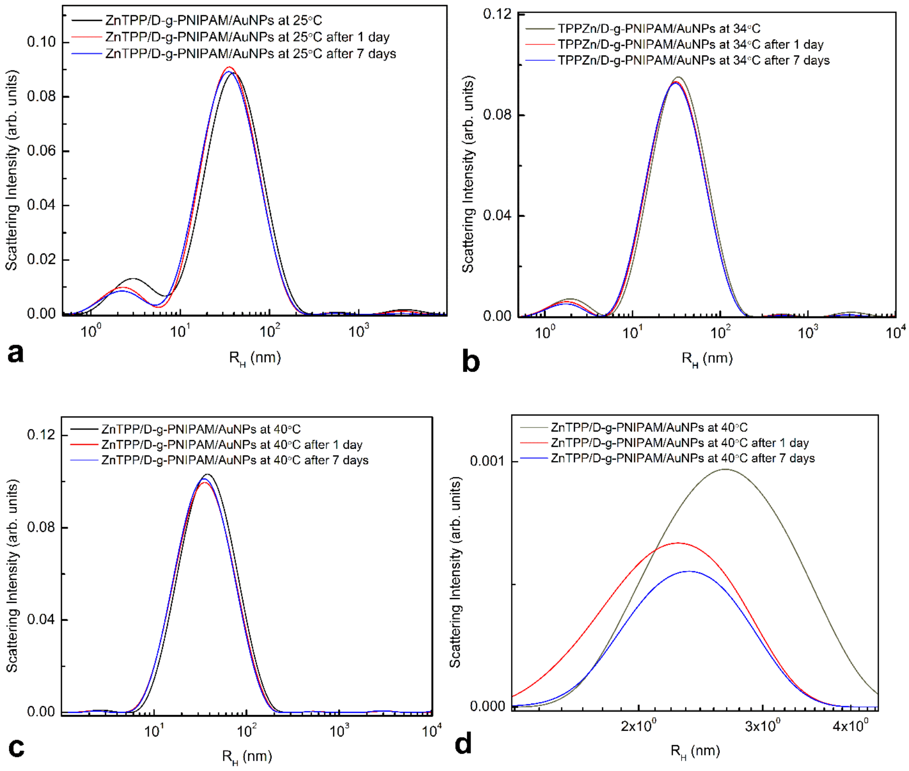

Figure S1. PSD of ZnTPP/D-g-PNIPAM/AuNPs fresh solution and one stored during 1 day and 7 days measured at 25 °C (**a**), 34 °C (**b**) and 40 °C (**c**); zoom-in of the first peak on the previous image (**d**).

2. Transformation of Absorption Spectrum of ZnTPP in Water and Aqueous Solutions of Hybrid Nanosystems

The Q bands and B (Soret) band are caused by $S_0 \rightarrow S_1$ and $S_0 \rightarrow S_2$ transitions from the π electron ground state to the 1st and 2nd excited π electron states of the porphyrin molecule, respectively. Q and B bands have a multicomponent structure that reflects the vibrational structure of excited electronic states. Transition $S_0 \rightarrow S_1$ is weakly allowed, while transition $S_0 \rightarrow S_2$ is strongly allowed by the selection rules, which causes a low intensity of the Q band and a high intensity of the B band in the spectrum.

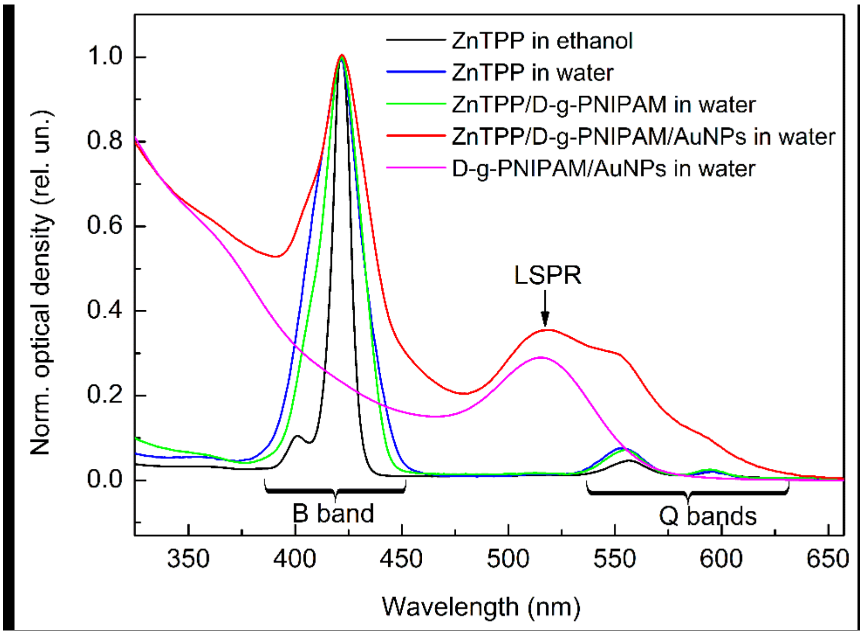

Figure S2. Normalized absorption spectra of ZnTPP in ethanol, ZnTPP in ethanol mixed with water, as well as ZnTPP/D-g-PNIPAM, ZnTPP/D-g-PNIPAM/AuNPs and D-g-PNIPAM/AuNPs aqueous solutions. The concentrations: ZnTPP – 0.001 g/L, D-g-PNIPAM – 0.078 g/L, Au – 0.077 g/L.

Mixing ethanol solution of ZnTPP with water leads to significant changes in the intensity and shape of the absorption spectrum, Figure S2. In Figure S2, spectra are normalized by intensity to better see the changes in the shape of the spectrum that occur during mixing. Since Figures S2 presents the normalized absorption spectra, it is not seen the change in the intensity, only in the shape of absorption spectrum there. Meanwhile, due to mixing, the total intensity of the B band decreases sharply by 2.5 times. At the same time, the B band essentially broadens and its doublet structure is blurred. Simultaneously, the intensity of the weak Q bands increases. Because ZnTPP is insoluble in water, it is likely that changes in the absorption spectrum are due to the aggregation of hydrophobic ZnTPP molecules in the aqueous medium. Assumptions about the aggregation of ZnTPP are fully consistent with the DLS data, which show the existence in water of aggregates of ZnTPP molecules with a radius of 32 nm, Figure 2b. It should be noted, however, that mixing ZnTPP ethanol solution with aqueous solutions of D-g-PNIPAM and especially with D-g-PNIPAM/AuNPs leads to reverse changes in absorption spectrum. Namely, the B band narrows that results in better visibility of its fine structure. Also, the B band intensity increases and the intensity of Q bands decreases. Herewith, the magnitude of the effect increases with increasing concentration of D-g-PNIPAM and with D-g-PNIPAM/AuNPs. This is probably due to the fact that the binding of ZnTPP molecules to D-g-PNIPAM polymer, and especially to D-g-PNIPAM/AuNPs nanohybrid, increases the solubility of ZnTPP in water. The increase in solubility should lead to a decrease in the size of ZnTPP aggregates, as evidenced by the results of DLS, Figure 3a.

3. Transformation of FL Spectrum of ZnTPP in Water and Aqueous Solutions of Hybrid Nanosystems

Bands F_{00} and F_{01} are caused by radiative transitions from the ground vibrational level of the 1st excited electronic state to the ground and the 1st excited vibrational levels of the ground electronic state of the porphyrin molecule [56,57].

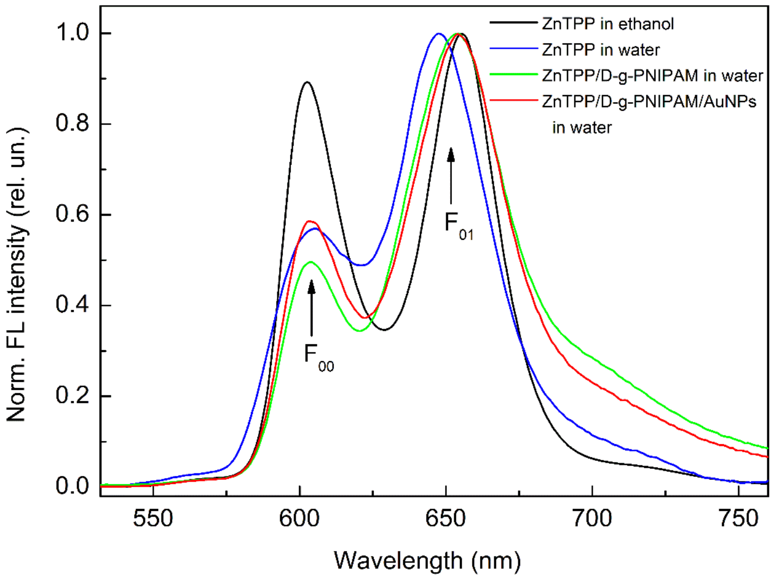

Figure S3. Normalized fluorescence spectra of ZnTPP in ethanol, in ethanol mixed with water, in ZnTPP/D-g-PNIPAM aqueous solution and ZnTPP/D-g-PNIPAM/AuNPs aqueous solution. Excitation: 421 nm; concentrations: ZnTPP – 0.001 g/L, D-g-PNIPAM – 0.078 g/L, Au – 0.077 g/L.

Similar to changes in the absorption spectrum, mixing ZnTPP with water leads to significant changes in the intensity and shape of the FL spectrum, Figure S3, blue line. In Figure S3, spectra are normalized by intensity to better see the changes in the shape of the spectrum that occur during mixing. Since Figure S3 presents the normalized FL spectra, it is not seen the change in the intensity, only in the shape of FL spectrum there. The total FL intensity decreases sharply by 8.5 times. The intensity of F_{00} band decreases significantly stronger compared to the intensity of F_{01} band. The long-wavelength F_{01} band shifts by 9 nm. Because ZnTPP is insoluble in water, as noted above, it is likely that the observed changes in the FL spectrum are associated with the aggregation of hydrophobic ZnTPP molecules in aqueous media. Further, mixing ZnTPP with an aqueous solution of polymer D-g-PNIPAM leads to a further decrease in the intensity of the F_{00} band compared to the case of mixing ZnTPP with water, Figure S3 (green line). However, the spectral position of the maximum of F_{01} band shifts back to the position of this band in the case of ethanol solution. Finally, the FL spectrum of the ZnTPP/D-g-PNIPAM/AuNPs mixture is transformed compared to the ZnTPP spectrum in ethanol, but these transformations are significantly weaker than for ZnTPP in water and ZnTPP in aqueous solution of D-g-PNIPAM. Thus, one can see that likely to changes in absorption spectrum, mixing ZnTPP ethanol solution with aqueous solutions of D-g-PNIPAM and especially with D-g-PNIPAM/AuNPs leads to reverse changes in FL spectrum. It is noted above that most probably it reflects the fact that the binding of ZnTPP molecules to D-g-PNIPAM polymer, and especially to D-g-PNIPAM/AuNPs nanohybrids, increases the solubility of ZnTPP in water that prevents the formation of ZnTPP aggregates.

4. Remarks on the Reabsorption Effect on Concentration Dependence of FL

The effect of polymer and Au concentration on ZnTPP FL intensity in ZnTPP/D-g-PNIPAM and ZnTPP/D-g-PNIPAM/AuNPs nanosystems was investigated, Figure 5. The

FL intensity at different concentrations was normalized by the FL intensity of ZnTPP in water. Since the FL spectrum of ZnTPP and the absorption spectrum of Au NPs overlap significantly (Figures S2 and S3), Au NPs absorb partially the fluorescence photons emitted by ZnTPP molecules. This leads to an attenuation of the signal when measuring the FL intensity. Therefore, the real FL intensity was calculated as $I_0(\lambda)=10^{D(\lambda)}I(\lambda)$, where $I_0(\lambda)$ is the real intensity of FL emitted by ZnTPP molecules and $D(\lambda)$ is the optical density of the sample caused by the light absorption by Au NPs. Here, all values are taken at the appropriate concentrations of gold. The respective normalized concentration dependence of FL intensity for ZnTPP/D-g-PNIPAM/AuNPs system is presented in Figure 4 by circles.

5. Photodynamic Antibacterial Activity in Vitro

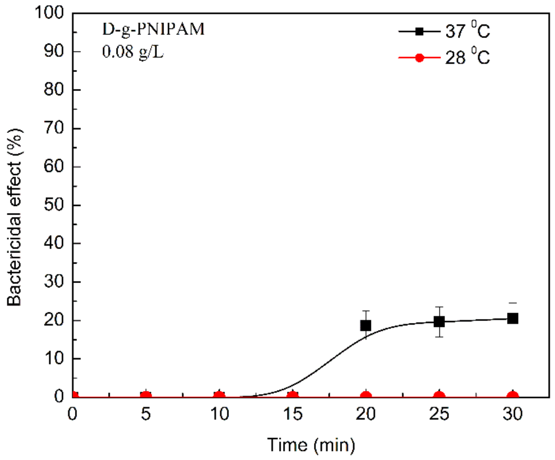

Figure S4. Antibacterial activity of D-g-PNIPAM 0.08 g/L at 28 °C and 37 °C.

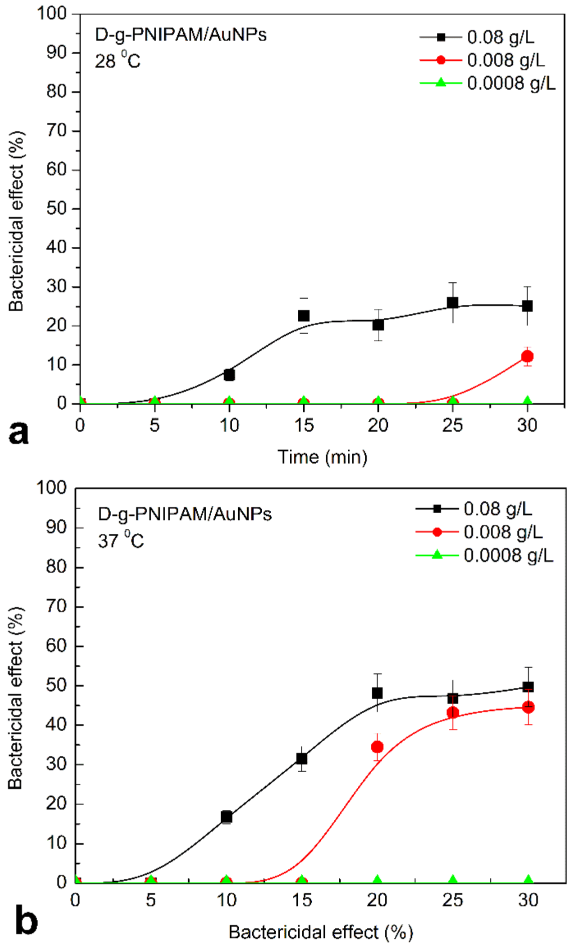

Figure S5. Inactivation of *S. aureus* by D-g-PNIPAM/AuNPs at 28 °C (**a**) and 37 °C (**b**). AuNPs concentrations: 0.08 g/L, 0.008 g/L, 0.0008 g/L.

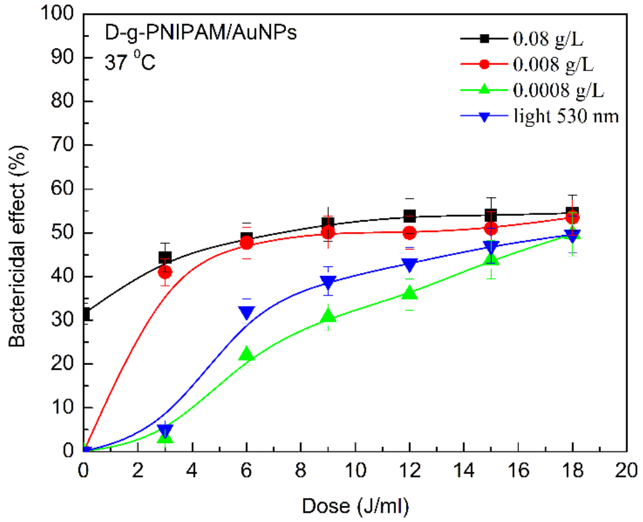

Figure S6. Inactivation of *S. aureus* in suspension at 37 °C after adding of D-g-PNIPAM/AuNPs and irradiation by light of 530 nm in dependence on the irradiation dose. AuNPs concentrations: 0.08 g/L, 0.008 g/L, 0.0008 g/L. Light power was 0.1 J/s, irradiation dose was in the range of 3–18 J/mL with increment of 3 J/mL.

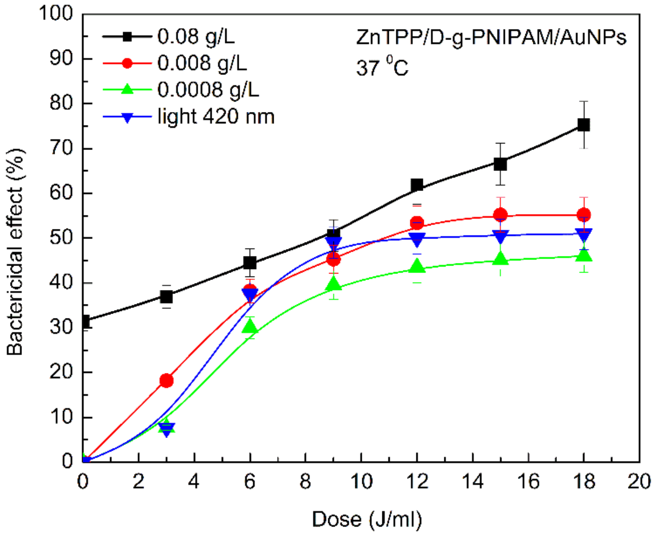

Figure S7. Inactivation of *S. aureus* in suspension at 37 °C after adding of ZnTPP/D-g-PNIPAM/AuNPs and irradiation by light at 420 nm in dependence on the irradiation dose. Concentrations: AuNPs – 0.08 g/L, 0.008 g/L, 0.0008 g/L, ZnTPP – 0.001 g/L. Light power was 0.1 J/s, irradiation dose was in the range of 3–18 J/mL with increments of 3 J/mL.

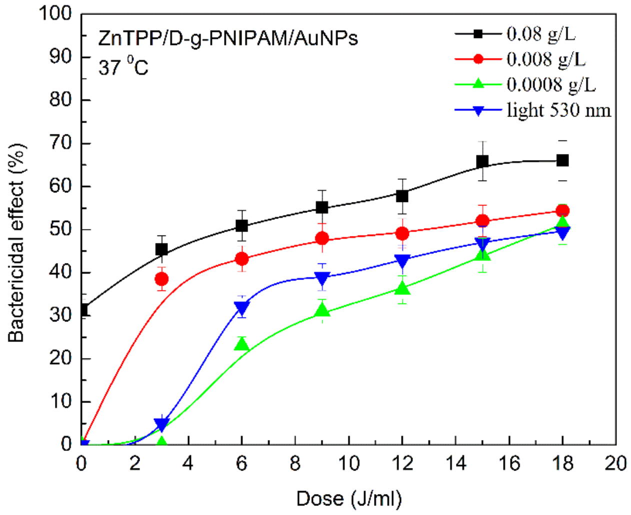

Figure S8. Inactivation of *S. aureus* in suspension at 37 °C after adding of ZnTPP/D-g-PNIPAM/AuNPs and irradiation by light at 530 nm depending on the irradiation dose. Concentrations: AuNPs – 0.08 g/L, 0.008 g/L, 0.0008 g/L, ZnTPP – 0.001 g/L. Light power was 0.1 J/s, irradiation dose was in the range of 3–18 J/mL with increment of 3 J/mL.
